# Psychosocial determinants of sexual satisfaction in older adults with multimorbidity: a cross-sectional study

**DOI:** 10.1186/s12877-026-07720-3

**Published:** 2026-05-30

**Authors:** Mirary Mantilla-Morrón, Aida Marina Ferrer Parejo, Damaris Suárez Palacio, Luz Mery Noguera, María Victoria Quintero-Cruz, Yuliana Martínez Gómez

**Affiliations:** 1 Universidad Simón Bolívar, Facultad de Ciencias de la Salud, Programa de Doctorado en Ciencias Clínicas, Centro de Investigaciones en Ciencias de la Vida, Universidad Simón Bolívar, Barranquilla, Colombia; 2 Universidad Simón Bolívar, Facultad de Ciencias de la Salud, Programa de Fisioterapia, Centro de Investigaciones en Ciencias de la Vida, Universidad Simón Bolívar, Barranquilla, Colombia; 3 Universidad Simón Bolívar, Facultad de Ciencias de la Salud, Programa de Fisioterapia, Centro de Investigación e Innovación Social José Consuegra Higgins, Universidad Simón Bolívar, Barranquilla, Colombia

**Keywords:** Aged, Multimorbidity, Sexual satisfaction, Quality of life, Selective disinvestment

## Abstract

**Background:**

Multimorbidity in later life transcends physical impairment, profoundly impacting subjective dimensions of well-being; however, the specific role of psychosocial factors in sexual health remains poorly understood. This study aimed to determine the independent associations between psychosocial quality of life domains, functional capacity, and sexual satisfaction in older adults with multimorbidity, adopting an integrated biopsychosocial perspective.

**Methods:**

A cross-sectional analytical study was conducted with 100 community-dwelling older adults (≥ 60 years) in Barranquilla, Colombia. Psychosocial QoL was assessed via the CASP-12 scale, functional capacity through the Duke Activity Status Index (DASI), and sexual satisfaction using the Index of Sexual Satisfaction (ISS). Diagnostic accuracy for multimorbidity was ensured through direct verification of medical records and prescriptions. Multivariate linear regression models with robust standard errors (HC3) and bootstrapping were employed to ensure statistical rigor.

**Results:**

The final analytical sample (N = 100) had a mean age of 72.6 pm 8.9 years, with a predominantly male composition (57.0%). The inclusion of psychosocial domains significantly enhanced the model’s explanatory power for sexual satisfaction (Final Adjusted R2 = 0.49; ∆ R2 = 0.21, p < 0.001). Autonomy (β = 12.07, p = 0.019) and Control (β = 10.19, p = 0.046) emerged essential positive pillars of satisfaction, while Self-realization exhibited a significant inverse association (β= -8.12, p=0.007). Male sex was independently associated with lower satisfaction scores. Regarding functional capacity (Adjusted R2 = 0.48), age and specific psychosocial domains were identified as key determinants of DASI performance.

**Conclusion:**

Psychosocial factors, particularly autonomy and control, significantly associated with sexual satisfaction in older adults with multimorbidity. These findings highlight the necessity of integrating mental health and subjective well-being into geriatric clinical care to support a holistic and humanized approach to healthy aging.

**Supplementary Information:**

The online version contains supplementary material available at https://doi.org/10.1186/s12877-026-07720-3.

## Introduction

The clinical landscape of aging is increasingly characterized by multimorbidity, defined as the co-occurrence of two or more chronic conditions in a single individual. While cardiovascular diseases (CVDs), hypertension, and type 2 diabetes remain the leading causes of global morbidity [1, 2], their impact on the older population is rarely isolated. In Latin America, the demographic transition has led to a high prevalence of individuals managing multiple simultaneous conditions, creating a ‘syndemic’ effect where diseases interact to exacerbate physical and psychological decline [3]. This clustering of conditions is often driven by shared underlying determinants, including metabolic risk factors, chronic inflammation, and lifestyle behaviors, all of which are deeply influenced by socioeconomic status [4, 5]. While the impact of these conditions on survival is well-documented, their influence on the subjective dimensions of well-being such as sexual health remains insufficiently understood in the context of multimorbidity [6].

Beyond traditional clinical parameters, psychosocial factors are fundamental to healthy aging and the effective management of multimorbidity. Social support, psychological well-being, and adaptive coping mechanisms directly influence health outcomes; for instance, depression is highly prevalent among patients with multiple chronic conditions, with its incidence escalating alongside disease severity. Adverse emotional states, including feelings of loss of control and diminished self-esteem, can exacerbate depressive symptoms, thereby compromising sexual satisfaction and overall quality of life (QOL) [7]. Social support acts as a vital buffer, where emotional and instrumental networks strengthen resilience and mitigate the anxiety associated with functional decline [8, 9]. In this context, resilience remains a critical determinant of QOL in later life [10]. Consequently, this study is grounded in the Biopsychosocial Model, which posits that health outcomes in multimorbidity are not merely the result of physiological pathways, but a complex interaction between biological burden, psychological resilience, and social context [8, 9]. Under this framework, sexual satisfaction is viewed as a multifaceted indicator of well-being that reflects the synergy between these domains.

Sexuality in later life is a core dimension of human experience, yet it is frequently neglected in clinical geriatric settings. It transcends mere genital function, encompassing intimacy, body image, and the need for emotional connection. Chronic diseases, including hypertension, T2DM, and obesity, profoundly impair functional capacity and have been linked to high rates of sexual dysfunction [11, 12]. Biologically, these conditions impose physical limitations including pain, fatigue, and dyspnea that restrict mobility and the physical energy required for sexual activity [13]. Evidence from both longitudinal and cross-sectional studies indicates that an increasing burden of physical illness is associated with diminished sexual satisfaction; in women aged 40 and older, each additional chronic condition significantly increases the likelihood of low libido [14, 15].

At a psychosocial level, sexual dysfunction is a common sequela of multimorbidity, correlating with physical health, mental status, and the quality of interpersonal relationships [14]. For example, chronic pain can diminish sexual desire and satisfaction, but these negative effects may be mitigated by robust social support [16]. In large-scale studies of older adults, poorer perceived health, depression, and pain have been consistently linked to sexual dissatisfaction; conversely, maintaining sexual desire is associated with better QOL and lower pain perception [17, 18]. Despite these insights, there is a gap in understanding how specific psychosocial domains interact in patients with multiple simultaneous conditions. This study aimed to determine the independent associations between psychosocial quality of life domains, functional capacity, and sexual satisfaction in older adults with multimorbidity, adopting an integrated biopsychosocial perspective.

## Methods

### Study design

A cross-sectional observational study was conducted following the STROBE (Strengthening the Reporting of Observational Studies in Epidemiology) guidelines. The study protocol was approved by the Research Ethics Committee of Universidad Simón Bolívar (No. CEI-USB-0451-00) and strictly adhered to the principles of the Declaration of Helsinki.

### Participants and setting

This study enrolled 100 community-dwelling (non-institutionalized) older adults in Barranquilla, Colombia. Recruitment took place through direct home visits in residential neighborhoods across the city between August 2023 and May 2024. Eligibility criteria comprised being 60 years of age or older, having a clinical diagnosis of multimorbidity (defined as the coexistence of two or more chronic conditions, such as hypertension and type 2 diabetes), and providing voluntary informed consent.

**Fig. 1:**
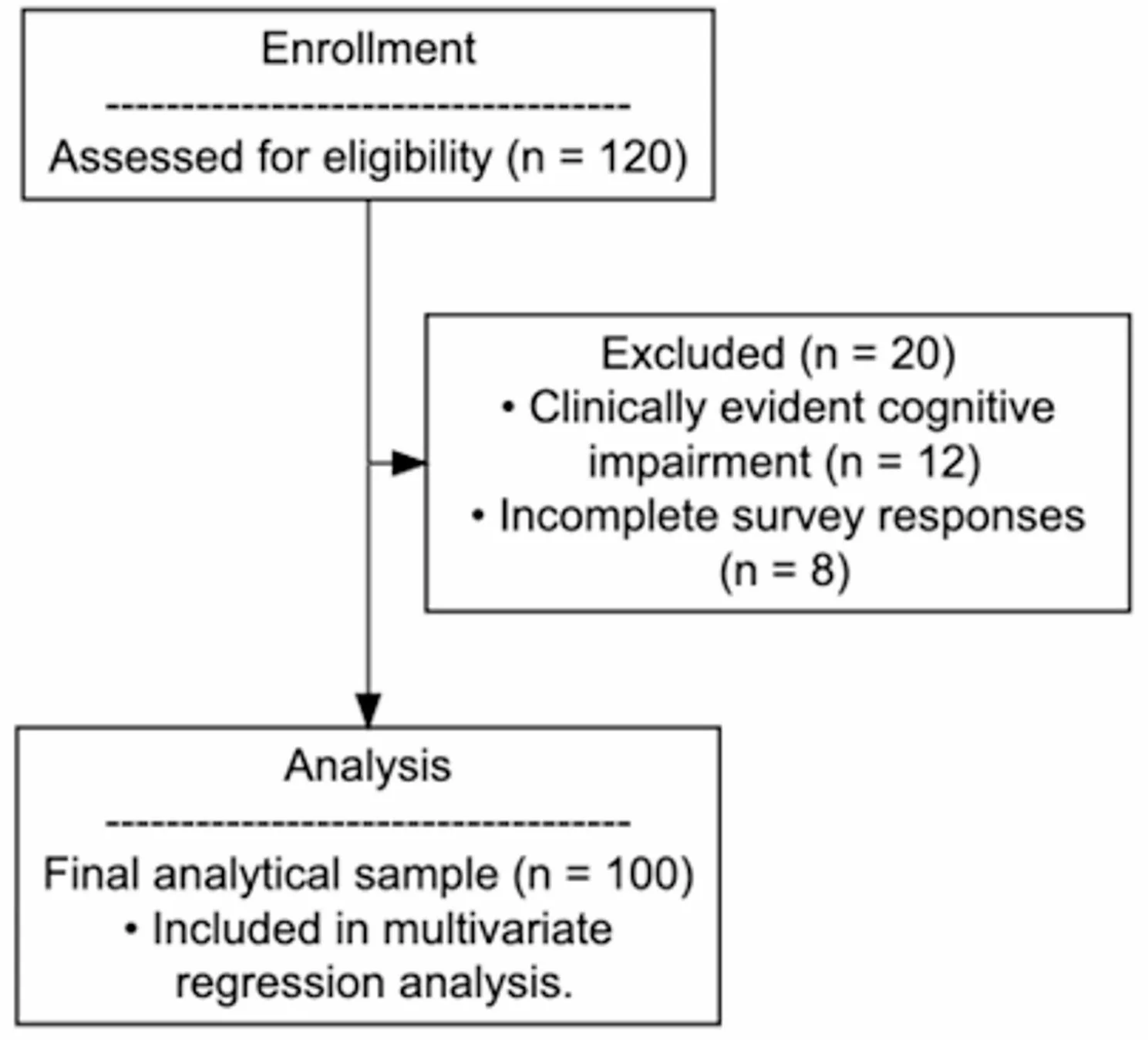
STROBE flow diagram of participant selection. Note: The diagram illustrates the selection process from initial screening (n = 120) to the final analytical sample (N = 100). Reasons for exclusion are categorized into clinically evident cognitive impairment and incomplete survey responses

Initially, 120 older adults were screened, as illustrated in the study flow diagram (Fig. 1). Of these, 100 met the eligibility criteria and were included in the final analytical sample (*N* = 100); 20 individuals were excluded due to clinically evident cognitive impairment (*n* = 12) or incomplete survey responses (*n* = 8). This sample size achieved a statistical power of 0.82 for a medium effect size (*f*^2^ = 0.15, α = 0.05).

### Data collection

Trained researchers performed recruitment and data collection through face-to-face home visits. To ensure diagnostic accuracy and address the verification of multimorbidity, clinical status was verified exclusively through the physical or digital presentation of medical records or pharmacological prescriptions during the visit. In all cases included in the analytical sample (*N* = 100), these documents were successfully accessed and verified; no participants were included based solely on self-reported diagnoses without documentary evidence. Interviews were conducted in private residential settings to ensure confidentiality and mitigate reporting bias.

### Exclusion criteria

Individuals were excluded if they had pre-existing cognitive impairment as diagnosed by a treating physician and reported by caregivers that prevented autonomous comprehension and completion of the research instruments. Additionally, individuals presenting with acute illness at the time of the interview, those with physical limitations preventing the interview, or those with incomplete clinical data were excluded.

### Measures

#### Sexual Satisfaction

Sexual satisfaction was assessed using a 4-item instrument adapted from the English Longitudinal Study of Ageing (ELSA) and its Sexual Relationships and Activities Questionnaire (SRA-Q) [19, 20]. This Spanish version captures the physical, emotional, and relational dimensions of sexual health and function in older adults. An Exploratory Factor Analysis (EFA) confirmed a robust single-factor structure (verified by KMO and Bartlett’s tests), while the scale demonstrated high internal consistency in this sample (Cronbach’s alpha = 0.80). The measure evaluates sexual desire, activity frequency, arousal capacity, and overall satisfaction. For clinical interpretability, raw scores were linearly transformed to a 0–100 metric via min-max normalization, with higher scores indicating greater satisfaction.

#### Functional capacity

Functional capacity was evaluated using the Spanish-validated Duke Activity Status Index (DASI) [21]. This 12-item self-report scale assesses the ability to perform activities of daily living that correlate with peak oxygen uptake (VO2 peak). The DASI has been widely utilized in Latin American geriatric research as a reliable proxy for physical independence and metabolic MET levels.

#### Psychosocial quality of life

The Spanish version of the CASP-12 scale was employed to evaluate psychosocial well-being [22]. This multidimensional instrument assesses four core domains: Control, Autonomy, Pleasure, and Self-realization. The use of this validated adaptation ensures that the linguistic nuances and the specific psychosocial context of the Colombian older population are accurately captured. In the present study, the scale’s internal reliability was further confirmed, consistent with previous geriatric cohorts.

### Statistical analysis

Data analysis was performed using R software (v.4.3.2) in two phases. First, the psychometric adequacy of the ISS was verified, confirming a robust unidimensional structure (KMO = 0.71; Bartlett’s *p* < 0.001) and high internal consistency (*α* = 0.82).

Second, multivariate linear regression models were estimated to identify predictors of sexual satisfaction. To ensure statistical rigor and address potential heteroscedasticity, Huber-White robust standard errors (HC3) were utilized, validated through bootstrap resampling (1,000 iterations). Multicollinearity was monitored via Variance Inflation Factors (VIF), with all values remaining below 1.4. The significance level was set at *p* < 0.05.

## Results

### Participant flow

The participant selection process and study flow are illustrated in the STROBE flow diagram (Fig. 1). Initially, 120 older adults were screened for eligibility. Of these, 100 met the inclusion criteria and were included in the final analytical sample (*N* = 100); 20 individuals were excluded due to clinically evident cognitive impairment (*n* = 12) or incomplete survey responses (*n* = 8).

### Descriptive data

The final sample had a mean age of 72.6 ± 8.9 years, with a distribution of 57% men and 43% women. Socioeconomic vulnerability was high, with 73% of participants in strata 1–2. The clinical profile of multimorbidity was led by arterial hypertension (55%), diabetes mellitus (22%), and obesity (12%). Despite these conditions, 65% reported being sexually active within the past year. Descriptive characteristics are further detailed in Table 1.

**Table 1:** Demographic, socioeconomic, and health characteristics of the study sample (*N* = 100)

Variable	Total (*N* = 100)	Male (*n* = 57)	Female (*n* = 43)	*p*-value
Demographics				
Age (years), mean ± SD	72.6 ± 8.9	73.1 ± 9.1	71.9 ± 8.7	0.512
Socioeconomic Status				
Low (Strata 1–2)	73 (73.0%)	42 (73.7%)	31 (72.1%)	0.856
Middle/Upper (Strata ≥ 3)	27 (27.0%)	15 (26.3%)	12 (27.9%)	
Health History (Comorbidities)				
Arterial Hypertension	55 (55.0%)	31 (54.4%)	24 (55.8%)	0.887
Diabetes Mellitus	22 (22.0%)	12 (21.1%)	10 (23.3%)	0.791
Obesity	12 (12.0%)	6 (10.5%)	6 (14.0%)	0.598
Asthma	9 (9.0%)	5 (8.8%)	4 (9.3%)	0.927
Sexual Health & Function				
Sexually Active (past year)	65 (65.0%)	38 (66.7%)	27 (62.8%)	0.690
Sexual Satisfaction (ISS)	**54.77 ± 23.67**	**46.8 ± 21.4**	**65.3 ± 22.1**	**< 0.01**
Functional Capacity (DASI)	19.2 ± 16.4	20.8 ± 17.1	17.1 ± 15.3	0.273

*p*-values are provided for descriptive and exploratory purposes to identify baseline distribution differences between sexes. These informed the selection of covariates for the multivariate regression models to ensure the stability and validity of the final estimates. Continuous variables were compared using Independent Samples T-tests, and categorical variables using Chi-square tests.

*SD* Standard deviation, *ISS* Index of Sexual Satisfaction, *DASI* Duke Activity Status Index

### Psychometric properties of the ISS

The structural validity of the Index of Sexual Satisfaction (ISS) was confirmed via Exploratory Factor Analysis (EFA), showing a robust unidimensional structure that explains 67.8% of the total variance. Sampling adequacy was deemed acceptable (KMO = 0.71; Bartlett’s *p* < 0.001), and internal consistency was excellent (*α* = 0.82). These metrics are provided in Supplementary Table 1.

### Main results: predictors of sexual satisfaction

The multivariate model for sexual satisfaction achieved a final adjusted *R*^2^ of 0.49, with psychosocial domains contributing a significant incremental variance (∆*R*^2^ = 0.21, *p* < 0.001). As shown in Table 4, higher Autonomy (β = 12.07, *p* = 0.019) and Control (β = 10.19, *p* = 0.046) were identified as essential pillars of satisfaction. Conversely, male sex (β = -12.01, *p* = 0.005) and Self-realization (β = -8.12, *p* = 0.007) showed significant negative associations.

### Secondary analysis: functional capacity

The model for functional capacity (DASI) yielded an adjusted *R*^2^ of 0.48. Control (β = -9.27, *p* < 0.001) and Autonomy (β = -6.64, *p* = 0.008) maintained significant associations with functional performance. Additionally, middle/upper socioeconomic status (β = 9.80, *p* = 0.015) and clinical conditions such as hypertension (β = 13.45, *p* < 0.001) were found to be associated with DASI variations.

### Sensitivity analysis and model diagnostics

To ensure the stability of the estimates, Variance Inflation Factors (VIF) were monitored, with all values remaining staying between 1.05 and 1.40, as detailed in in Supplementary Table. All models were validated using Huber-White robust standard errors (HC3) and bootstrap resampling (1,000 iterations) to mitigate the effects of heteroscedasticity and ensure precise reporting of the coefficients.

### Sociodemographic characteristics and health status

The participant selection process and study flow, following STROBE guidelines, are illustrated in Fig. 1. The final analytical sample (*N* = 100) had a mean age of 72.6 ± 8.9 years, with a slight male predominance (57.0%). As detailed in Table 1, the population reflects significant socioeconomic vulnerability, with 73.0% belonging to low-income strata (Strata 1–2).

Regarding health status, Table 1 reveals a high prevalence of chronic conditions, led by arterial hypertension (55.0%) and diabetes mellitus (22.0%). Despite this clinical burden, 65.0% of participants reported being sexually active within the past year. Furthermore, while psychosocial quality of life domains specifically Autonomy (2.84 ± 0.71) and Control (2.26 ± 0.74) suggest a preserved sense of agency, sexual satisfaction (ISS) scores exhibited wide variance (54.77 ± 23.67). These baseline differences, particularly when stratified by sex (*p* < 0.01), provide the empirical rationale for the multivariate modeling presented in subsequent sections.

### Multivariate regression models

Multivariate regression analysis indicated that psychosocial domains are significant predictors of sexual satisfaction, accounting for a substantial portion of the explained variance (Table 2). Specifically, higher levels of Autonomy and Control were independently associated with increased sexual satisfaction, whereas male sex and Self-realization emerged as significant negative predictors.

**Table 2:** Multivariate linear regression models for sexual satisfaction and functional capacity

Predictor	Model A: Sexual Satisfaction (ISS)		Model B: Functional Capacity (DASI)	
	β(SE)	*p*-value	β(SE)	*p*-value
Constant	45.12 (12.4)	< 0.001	68.34 (8.5)	< 0.001
Sociodemographics				
Age	-0.15 (0.24)	0.531	-0.63 (0.18)	0.001*
Sex (Male)	-12.01 (4.20)	0.005*	2.15 (3.10)	0.488
SES (Middle/Upper)	3.42 (5.10)	0.505	1.84 (4.25)	0.665
Psychosocial QoL (CASP-12)				
Control	10.19 (5.03)	0.046*	-9.27 (2.95)	0.002*
Autonomy	12.07 (5.05)	0.019*	-6.64 (2.49)	0.009*
Self-realization	-8.12 (2.94)	0.007*	-0.45 (2.12)	0.832
Pleasure	2.14 (3.15)	0.498	3.12 (2.45)	0.205
Model Diagnostics				
*R*^*2*^ (Adjusted *R*^*2*^)	0.25 (0.21)		0.32 (0.28)	
F-statistic	7.74	< 0.001	5.03	< 0.001

*β* Unstandardized coefficient, *SE* Robust Standard Error (HC3). Models validated via bootstrap resampling (1,000 iterations). *ISS* Index of Sexual Satisfaction, *DASI* Duke Activity Status Index

*Significant at p < 0.05

Regarding functional capacity, the model demonstrated a robust fit. Advanced age, alongside the psychosocial domains of Control and Autonomy, showed significant negative associations with DASI scores. Diagnostic metrics, including Variance Inflation Factors (VIF < 1.4) and bootstrap resampling (1,000 iterations), confirmed the stability and robustness of these estimates, with no evidence of multicollinearity.

## Discussion

This study investigated the psychosocial determinants of sexual satisfaction and functional capacity in a cohort of older adults with multimorbidity. Our findings indicate that beyond physical health, the domains of Autonomy and Control fuction as pivotal predictors of sexual well-being. This aligns with the biopsychosocial model and the perspective of Træen and Villar [20], suggesting that personal agency and self-determination may be as critical as clinical status in later life [18, 20]. Although sexuality in older age is frequently influenced by the confluence of senescence and chronic disease, psychosocial determinants profoundly shape the perception and meaning of sexual life in this population [21–23].

The community-based nature of this study, involving interviews conducted in participants’ private residences in Barranquilla, offers a distinct perspective compared to clinical samples. This setting potentially facilitated a more transparent dialogue regarding intimate topics, reflecting real-world experiences. Such a context captures the perspectives of older adults who are not necessarily seeking medical intervention for sexual dysfunction but are navigating their sexuality within the constraints of daily life and low socioeconomic status.

The high prevalence of hypertension and diabetes in our sample reflects the ‘clinical burden’ described in international literature, where multimorbidity transcends the impact of single diseases on sexual function [14, 24]. Interestingly, our results showed that male sex was independently associated with lower sexual satisfaction. While European studies like ELSA [19, 20] often report lower satisfaction in women due to physiological changes, our findings in a Colombian Caribbean context suggest a different dynamic. In Barranquilla, where traditional ‘machismo’ and the role of the male as a provider remain culturally significant, the onset of multimorbidity may be perceived as a threat to masculine identity. This ‘masculinity crisis’ associated with physical decline in socioeconomically vulnerable environments could explain why men in our sample reported lower satisfaction, a phenomenon also observed by Pérez Aranda et al. in other Ibero-American populations [24, 25]. Furthermore, as noted by Shen [ chronic pain may diminish sexual desire and satisfaction through its interplay with stress and the altered body image associated with chronic disease [26].

One of the most relevant findings is the inverse association between Self-realization and sexual satisfaction, this finding challenges the typical expectation of positive correlations among well-being domains. This paradox may be interpreted through the Selective Disinvestment Hypothesis and the Selective Optimization with Compensation (SOC) model [27, 28]. As proposed by Wrosch et al. [28], older adults with multimorbidity may shift their cognitive and emotional resources toward goals perceived as more attainable such as family relationships or spiritual peace reducing their investment in sexuality when it becomes physically constrained or conflictual [15, 16, 24]. While this adaptive ‘disinvestment’ has been frequently documented in female cohorts [20–22], our findings reflecting a majority-male sample (57%) suggest that for men, this transition might be more conflictual. In the cultural context of the Colombian Caribbean, masculine self-realization is often traditionally tied to physical and sexual agency, which may explain why higher scores in other life domains do not positively correlate with sexual satisfaction, but rather highlight the perceived loss of sexual well-being.

Similarly, we observed that higher autonomy was inversely related to self-reported functional capacity (DASI). We propose the functional awareness paradox, wherein highly autonomous individuals may apply more critical self-evaluation criteria to their physical performance. This leads to a more acute perception of their limitations without necessarily reflecting reduced resilience, a trend consistent with prior evidence from Paz et al. [29] and Reis Júnior [9] regarding functional dependence and its impact on family roles in Latin America [28, 29].

Our results regarding functional capacity are consistent with national data from the SABE Colombia Study [30], which highlights how chronic conditions and low income converge to affect the independence of the Colombian elderly. Furthermore, the correlation between better perceived health and sexual desire mirrors findings from Rocamora-Pérez et al. [31], reinforcing the idea that sexual health is a proxy for general well-being. The positive influence of social support and the absence of depression as protective factors is supported by Acoba [8] and Hu et al. [17]. Emotional and instrumental networks appear to mitigate the stress of disability and chronic pain [31]. In the Colombian context, where family networks are primary providers of care, this support becomes a critical buffer for maintaining quality of life despite multimorbidity [29, 30]. Ultimately, while physical health influences sexual activity, psychorelational mechanisms including intimacy redefinition and expectation recalibration likely play a central role independent of morbidity [32–35].

### Study limitations

Several limitations should be considered when interpreting these findings. First, the cross-sectional design precludes the establishment of causal relationships between psychosocial factors and sexual satisfaction; therefore, our results should be viewed as significant associations rather than directional effects. Second, the sample (*N* = 100) was obtained through non-probability sampling in a specific urban context (Barranquilla, Colombia), which may limit the generalizability of the findings to broader populations. However, the high ecological validity of home-based recruitment reaching older adults in their natural environments and the application of bootstrapping (1,000 resamples) were intended to strengthen the reliability of our predictive models.

Additionally, all primary variables relied on self-reported data, which may introduce social desirability or recall bias, particularly regarding sensitive and deeply personal topics such as sexual health. Functional capacity was estimated using the DASI rather than direct physiological measurements, which, while a validated proxy, may lack the precision of objective metabolic testing. Furthermore, although robust standard errors (HC3) were employed to ensure statistical rigor, we cannot entirely rule out the influence of unmeasured confounding factors such as the health status of a partner or the specific effects of certain medications on sexual function. Finally, while the ISS demonstrated strong internal consistency in this cohort, further validation in larger and more diverse groups would be beneficial to confirm its robustness across different cultural and clinical settings.

## Conclusions

In the experience of older adults living with multimorbidity, autonomy and a sense of control over one’s life emerge as essential pillars of sexual satisfaction, potentially holding more significance than the physical impact of their chronic conditions. A thought-provoking finding is the possible selective sexual disinvestment suggested by the inverse association between self-realization and sexual satisfaction. This suggests that as physical constraints increase, individuals may naturally reorient their energy and affection toward other meaningful life domains; however, this remains an interpretative hypothesis that should be explored more deeply through longitudinal studies to understand how these dynamics evolve over time.

These results highlight the value of adopting a holistic gaze in geriatric care one that moves beyond treating diseases to embrace emotional well-being and intimacy as fundamental components of healthy aging. While this study serves as an initial exploration within a specific geographical context, it provides a meaningful starting point for future research to further clarify how the well-being of the spirit and physical health are intertwined in later life. 

## Supplementary Information

Supplementary Material 1. Exploratory Factor Analysis (EFA) for the ISS.

Supplementary Material 2. Variance Inflation Factors for Predictors in Multivariate Regression Models.

## Data Availability

Due to the sensitive nature of sexual health data and confidentiality restrictions established in the ethics approval, the dataset cannot be deposited in a public repository. Fully anonymized data may be made available upon reasonable request to the corresponding author.
