# Supplementary material for: Psychosocial determinants of sexual satisfaction in older adults with multimorbidity: a cross-sectional study
Source: BMC Geriatr. 2026 May 30;26:1164. doi: 10.1186/s12877-026-07720-3 (PMC13576093; doi:10.1186/s12877-026-07720-3)
Supplement: Supplementary file 1 [file 12877_2026_7720_MOESM1_ESM.docx]

**Supplementary Table 1: Exploratory Factor Analysis (EFA) for the Index of Sexual Satisfaction (ISS)**

Psychometric Properties and Model Fit

| Psychometric Parameter | Value | Interpretation |
| --- | --- | --- |
| Kaiser-Meyer-Olkin (KMO) Measure of Sampling Adequacy | 0.710 | **Acceptable.** Indicates that the item correlations are sufficient for an EFA. |
| Bartlett's Test of Sphericity  (*p*-value) | *χ*2=196.22 (*gl*=6) | Significant (*p* < 0.001), justifying the presence of meaningful correlations. |
| Variance Explained by Factor 1 | 67.8% | Solid Unidimensionality. A single factor accounts for the majority of the total variance. |
| Internal Consistency (Cronbach's α) | 0.82 | Very good internal consistency. |
| Eigenvalue (λ1) | 2.71 | Retained Factor. The eigenvalue exceeds the Kaiser criterion (λ>1), confirming a single-factor structure. |

**Factor Loadings and Communalities**

| ****Index of Sexual Satisfaction (ISS) Item**** | Factor Loading | Communality (*h*) |
| --- | --- | --- |
| Capacity to become sexually aroused | **−0.940** | 0.884 |
| Level of sexual desire | **−0.868** | 0.754 |
| Frequency of sexual activities | **−0.766** | 0.587 |
| Satisfaction with sexual life in general | **−0.697** | 0.486 |

***Note:*** *The negative signs are due to the original coding direction of the items in the database; interpretation of the magnitude is based on the absolute value of the loading, with all values being ≥0.69 (high loadings).*

*The presence of negative factor loadings (e.g., -0.940) is standard in psychometric analysis. This simply indicates that the scale direction of the item (e.g., scoring high on 'arousal capacity') runs counter to the scoring direction of the latent factor. This commonly occurs when items are not reverse-coded prior to EFA or when question phrasing varies. Given that the internal consistency (Cronbach's* α *= 0.82) is excellent, this confirms the scale is highly reliable and the negative loadings are purely structural. The model remains fully valid provided the final interpretation accounts for this scaling alignment.*
