# Supplementary material for: Psychosocial determinants of sexual satisfaction in older adults with multimorbidity: a cross-sectional study
Source: BMC Geriatr. 2026 May 30;26:1164. doi: 10.1186/s12877-026-07720-3 (PMC13576093; doi:10.1186/s12877-026-07720-3)
Supplement: Supplementary file 2 [file 12877_2026_7720_MOESM2_ESM.docx]

**Supplementary Table 2: Variance Inflation Factors (VIF) for Predictors in Multivariate Regression Models**

Description: Variance Inflation Factors (VIF) calculated for the predictors included in Model A (Sexual Satisfaction – ISS) and Model B (Functional Capacity – DUKE-DASI). All VIF values remained within a strict range between 1.05 and 1.40, confirming the complete absence of problematic multicollinearity and ensuring the stability of the estimated regression coefficients**.**

| Variable | VIF (Model A: ISS) | VIF (Model B: DUKE-DASI) |
| --- | --- | --- |
| Age | 1.17 | 1.17 |
| Sex (Male) | 1.17 | 1.17 |
| SES (Middle/upper) | 1.28 | 1.28 |
| Control (CASP-12) | 1.05 | 1.05 |
| Autonomy (CASP-12) | 1.24 | 1.24 |
| Pleasure (CASP-12) | 1.09 | 1.09 |
| Self-realization (CASP-12) | 1.40 | 1.40 |
| Hypertensión | 1.22 | 1.22 |
| Obesity | 1.13 | 1.13 |
| Diabetes | 1.15 | 1.15 |

***Note:*** *All VIF values are < 5.0, confirming the absence of multicollinearity. (SES: Socioeconomic Status; ISS: Index of Sexual Satisfaction; DASI: Duke Activity Status Index).*
